# Molecular cloning and characterization of *amh* and *dax1* genes and their expression during sex inversion in rice-field eel *Monopterus albus*

**DOI:** 10.1038/srep16667

**Published:** 2015-11-18

**Authors:** Qing Hu, Wei Guo, Yu Gao, Rong Tang, Dapeng Li

**Affiliations:** 1 College of Fisheries, Huazhong Agricultural University, Wuhan 430070, China; 2 Life Science College, Hunan University of Arts and Science, Changde 415000, China; 3 Freshwater Aquaculture Collaborative Innovation Center of Hubei Province, Wuhan 430070, China; 4 Key Laboratory of Freshwater Animal Breeding, Ministry of Agriculture, Wuhan 430070, China

## Abstract

The full-length cDNAs of *amh* and *dax1* in the hermaphrodite, rice-field eel (*Monopterus albus*), were cloned and characterized in this study. Multiple sequence alignment revealed Dax1 was well conserved among vertebrates, whereas Amh had a low degree of similarity between different vertebrates. Their expression profiles in gonads during the course of sex inversion and tissues were investigated. The tissue distribution indicated *amh* was expressed mostly in gonads and was scarcely detectable in other tissues, whereas the expression of *dax1* was widespread among the different tissues, especially liver and gonads. *amh* was scarcely detectable in ovaries whereas it was abundantly expressed in both ovotestis and testis. By contrast, *dax1* was highly expressed in ovaries, especially in ♀IV (ovaries in IV stage), but it was decreased significantly in ♀/♂I (ovotestis in I stage). Its expression was increased again in ♀/♂III (ovotestis in III stage), and then decreased to a low level in testis. These significant different expression patterns of *amh* and *dax1* suggest the increase of *amh* expression and the decline of *dax1* expression are important for the activation of testis development, and the high level of *amh* and a low level of *dax1* expression are necessary for maintenance of testis function.

Rice-field eel (*Monopterus albus*) is a species of the teleost family Synbranchidae of the order Synbranchiformes (Neoteleostei, Teleostei, Vertebrata). This freshwater fish, which is an important species for fishery production, is a good model for comparative genomic studies of vertebrates, especially for sexual development owing to its special characteristics, including a relatively small genome compared to other teleosts and natural sex inversion (from female to male via intersex) during its life cycle^1^,^2^.

Anti-Müllerian hormone (AMH), also called Müllerian-inhibiting substance (MIS), which encodes a glycoprotein member of the transforming growth factor-β (TGF-β) superfamily^3^,^4^, is a gonadal hormone that initializes the regression of Müllerian ducts. Müllerian ducts in mammals are characteristic reproductive structures of the female that would differentiate into fallopian tubes and uterus^5^,^6^. In males, *Amh* is one of the first genes to be expressed strongly in Sertoli cells and causes regression of the Müllerian ducts during testicular differentiation^7^. AMH prevents the FSH-stimulated aromatase activity in Sertoli cell. It also inhibits the testosterone production stimulated by LH in cultured fetal Leydig cells in immature testis^8^. AMH dramatically decreases the conversion rate of testosterone to estradiol through reducing the activity of aromatase when ovine fetal ovaries exposed to AMH^9^. AMH acts mainly through the Amh/AmhrII system in gonad differentiation and development^10^,^11^. Müllerian ducts develop in only a few species of fish, such as sturgeon (*Acipenser* spp.), without undergoing regression in males^12^. *amh* orthologs, however, have been identified in various teleost species, including *Paralichthys olivaceus* (Japanese flounder)^13^, *Danio rerio* (Zebrafish)^14^, *Oryzias latipes* (Medaka)^15^, *Dicentrarchus labrax* (European seabass)^16^, *Squalius alburnoides* (Bordalo)^17^, *Acanthopagrus schlegeli* (Black Porgy)^18^ and *Carassius auratus* var. Pengze (Pengze crucian carp)^19^. It was also involved in sex differentiation and development in these species.

DAX1 (dosage-sensitive sex reversal, adrenal hypoplasia congenita, critical region on the X-chromosome, gene 1), also called NR0B1, is a member of an unusual orphan nuclear receptor superfamily^20^. In general, it contains a typical ligand-binding domain (LDB) in the C-terminal region and a characteristic DNA-binding domain (DBD) in the N-terminal region^21^. DAX1 would be able to modulate the transcription of several genes involved in the development of the adrenal and gonadal tissues, and steroidogenic activity, such as estrogen receptors α and β^22^, androgen receptor^23^, *Amh*^24^, *Cyp19*^25^ and *CYP17*^26^. Over-expression of *Dax1* induced male to female sex inversion in a normal male mouse^20^,^27^. *Dax1* was also shown to be essential for normal testicular development and influences male fertility in mice^28^. *dax1* is a conservative gene and its orthologs have been cloned in fish, including *Oreochromis niloticus* (Nile tilapia)^29^, *D. labrax*^30^ and *O. latipes*^31^. Recent studies showed *dax1* might participate in gonad differentiation and development.

In the hermaphrodite fish *M. albus*, only a few genes potentially participating in sex inversion have ever been isolated, and there is a paucity of knowledge about the mechanisms of sex inversion. *amh* is one of the genes reported to participate actively in the pathway of male sex determination. *dax1* might be involved in gonad differentiation and development in both sexes of several teleost species. Taking into account the function of *amh* and *dax1* in sex inversion of *M. albus* is not fully understood, we cloned the full-length cDNA of *amh* and *dax1* and analyzed their patterns of expression in different periods during sex inversion, as well as their tissue distributions. With this approach, we aimed to elucidate the role of these genes in gonad development and provide insight into the mechanism underlying sex inversion in *M. albus*.

## Results

### Cloning and sequence analysis of *amh* and *dax1*

**Figure 1:**
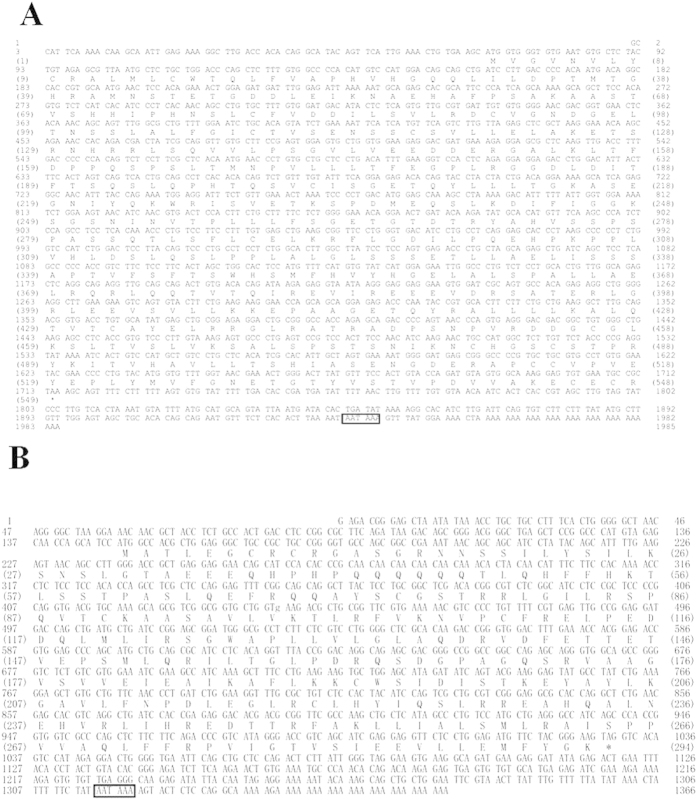
Nucleotide and deduced amino acid sequences for *M. albus amh* (**A**) and *dax1* (**B**).The amino acid sequence is shown in standard 1-letter code below the nucleotide sequence. The box indicates the typical polyadenylation signal. The positions of amino acid residues are given in parentheses.

The full-length cDNAs of *M. albus amh* (KF770790.1) and *dax1* (KF770791.1) are 1985 bp and 1366 bp, respectively, and the corresponding open reading frames (ORFs) are 1647 bp and 882 bp, which encode 548 and 293 amino acids, respectively. The 5′ untranslated regions (5′-UTRs) of *amh* and *dax1* are 68 bp and 148 bp and the 3′-UTRs are 270 bp and 336 bp, respectively. All of them contain a putative polyadenylation signal (AATAAA) closely upstream of the poly(A) tail (Fig. 1).

**Figure 2:**
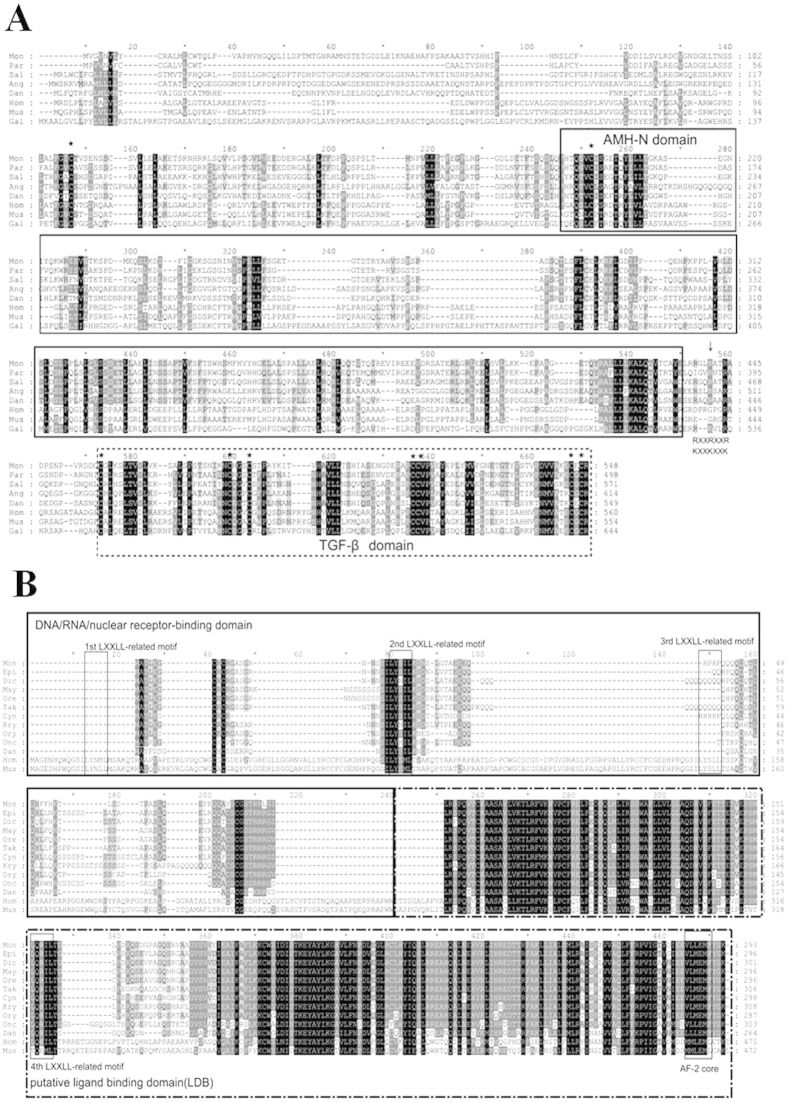
Amino acid sequence comparison of *M. albus* Amh (**A**) and Dax1 (**B**) with known orthologs.(**A**) The AMH domain (AMH-N in solid line box) that characterize this protein family are labeled and the TGF-β domain at the C-terminus (TGF-β in dotted line box). The asterisks indicated the conservative cysteines. Mon: *Monopterus albus*; Par: *Paralichthys olivaceus*, BAD37138.1; Sal: *Salmo salar*, AAU85130.1; Ang: *Anguilla japonica*, BAB93107.1; Dan: *Danio rerio*, AAX81416.1; Hom: *Homo sapiens*, AAH49194.1; Mus: *Mus musculus*, NP_031471.2; Gal: *Gallus gallus*, NP_990361.1. (**B**) Four LXXLL-like motifs were found in DAX1s (in the fine line boxes). Three of them are located in the DNA/RNA/nuclear receptor-binding domain (in unbroken line box). The fourth LXXLL-like motif and AF-2 core are located in the ligand-binding domain (in dotted line box). Mon: *Monopterus albus*; Epi: *Epinephelus coioides*, ADR80691.1; Dic: *Dicentrarchus labrax*, CAG17628.1; May: *Maylandia zebra*, XP_004551298.1; Ore: *Oreochromis niloticus*, AAN17672.1; Tak: *Takifugu rubripes*, XP_003961802.1; Cyn: *Cynoglossus semilaevis*, ACZ51263.1; Kry: *Kryptolebias marmoratus*, ACL00866.1; Ory: *Oryzias latipes*, BAF74811.1; Onc: *Oncorhynchus mykiss*, ACM80361.1; Dan: *Danio rerio*, AAI33943.1; Hom: *Homo sapiens*, ADZ17343.1; Mus: *Mus musculus*, NP_031456.1.

Multiple sequence alignment of Amh with all deduced protein sequences between *M. albus* and other vertebrates showed the level of conservation of Amh among different species was low; e.g. 64.3% between *M. albus* and *P. olivaceus*. A low level of similarity has been also detected between *M. albus* and *Mus musculus* (Mouse) (18.2%) and between *M. albus* and *Homo sapiens* (Human) (18.1%) (Fig. 2A). The C-terminal region shared a higher degree of identity among different species compared to the N-terminal regions. The amino acid sequence of Amh contains two domains characteristic of the AMH protein family, the AMH-N domain in the middle of this protein and the TGF-β domain at the C terminus. It showed the presence of two consecutive putative plasmin cleavage sites (R/K-XX-R/K) between these two domains (arrow in Fig. 2A). Finally, analysis of the cysteine distribution showed there are nine cysteine residues conserved among different vertebrates (asterisks in Fig. 2A).

Multiple alignment of the Dax1 deduced sequence showed the highest degree of identity between *M. albus* and *Epinephelus coioides* (Orange-spotted grouper) (89.8%). Furthermore, a higher level of conservation was found in the C-terminal region compared to the N-terminal regions. Four LXXLL-like motifs were found in mammalian DAX1 proteins; three were located in the DNA/RNA/nuclear receptor-binding domain and one was located in the ligand-binding domain. By contrast to mammals, teleost Dax1 has only two LXXLL-like motifs, one located in the DNA/RNA/nuclear receptor-binding domain and the other in the ligand-binding domain. Finally, an AF-2 core was found in the ligand-binding domain of mammals and fish (Fig. 2B).

**Figure 3:**
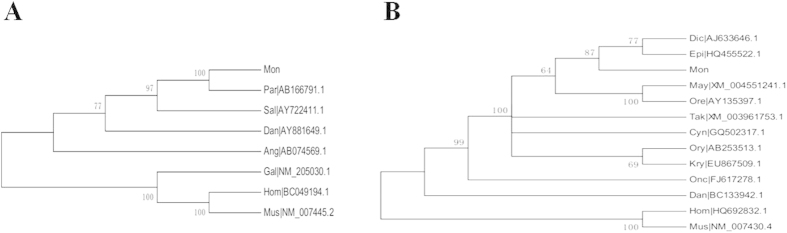
Phylogenetic tree based on *amh* (**A**) and *dax1* (**B**) nucleotide sequences. The abbreviations of species are the same as those used in Fig. 2. The numbers are the percentage of bootstrap values supporting each node from 1000 replicas on the basis of the neighbor-joining method.

Phylogenetic analysis of *amh* nucleotide sequences showed *M. albus* clusters within the clade of *P. olivaceus* with high bootstrap support (100), well separated from mammals and *Gallus gallus* (chicken) as an outgroup (Fig. 3A). A phylogenetic tree of *dax1* nucleotide sequences showed *M. albus* was related most closely to *D. labrax* and *E. coioides* (bootstrap 87) and was well separated from mammals as an outgroup (Fig. 3B).

### Tissue-specific distribution of *amh* and *dax1*

**Figure 4:**
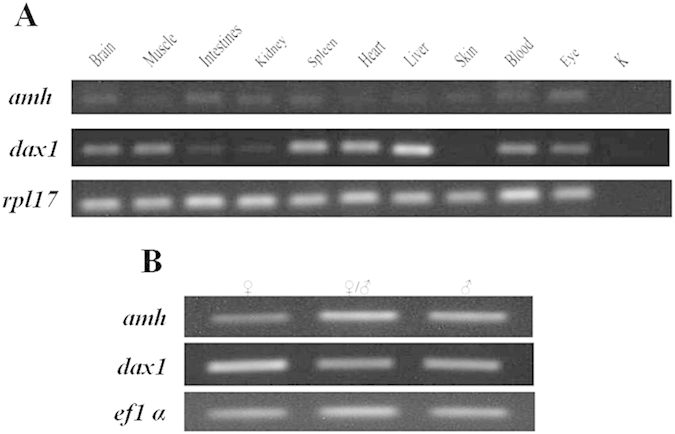
RT-PCR analysis of *amh* and *dax1* expression in various tissues of *M. albus*. (**A**) expression levels in different tissues, (**B**) expression levels in different development stages of gonads, K: negative control, *rpl17* and *ef1*α: internal controls.

*amh* and *dax1* were detected in brain, muscle, intestine, kidney, spleen, heart, liver, skin, blood, eye and gonads by RT-PCR, with the highest level in the gonads (Fig. 4). The transcript levels of *amh* were scarcely detectable in tissues other than gonads. By contrast, the level of *dax1* expression was highest in liver and gonads followed by heart, spleen, blood, muscle, brain and eye (Fig. 4A). Among the different development stages of gonads, the highest level of *amh* mRNA was observed in ovotestis and testis, whereas the highest level of *dax1* mRNA was detected in ovaries (Fig. 4B).

### Expression of *amh* and *dax1* during sex inversion of *M. albus*

**Figure 5:**
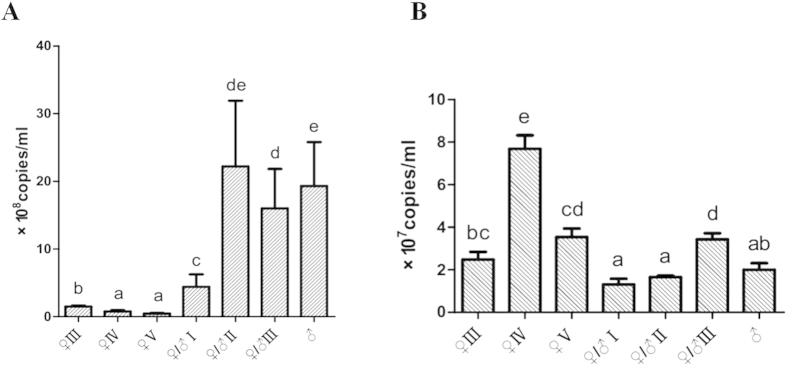
The copies of *amh* (**A**) and *dax1* (**B**) in different development stages of gonads in *M. albus*. ♀III: ovaries in III stage, ♀IV: ovaries in IV stage, ♀V: ovaries in V stage, ♀/♂I: ovotestis in I stage, ♀/♂II: ovotestis in II stage, ♀/♂III: ovotestis in III stage, ♂: testis. Two biological replicates have been used in each stage, and each samples being measured three times. Bars with the different letter indicate significantly difference from one another (Friedman ANOVA, *P* < 0.05). Data are given as mean ± SE. (**A**) *amh* expression significantly decreased when ovary developed from ♀III to ♀IV, and then significantly increased when the gonadal development changed into the ovotestis (Friedman ANOVA, *P* < 0.05). (**B**) The expression of *dax1* significantly increased when gonadal development entered into ♀IV from ♀III, and then significantly decreased when the gonadal developed from ♀V to ♀/♂I (Friedman ANOVA, *P* < 0.05).

The level of *amh* expression was down-regulated significantly when the gonad developed to ♀IV (ovaries in IV stage) from ♀III (ovaries in III stage). But it was up-regulated dramatically when the gonad inversed to ovotestis from ovaries, and the level of expression was very high throughout the ♀/♂II (ovotestis in II stage), ♀/♂III (ovotestis in III stage) and ♂ (testis) stages (Friedman ANOVA, *P* < 0.05; Fig. 5A). The expression of *dax1* appeared to be more stable compared to *amh* during the development stages of gonads. It was increased significantly during oocyte maturation (♀IV) (Friedman ANOVA, *P* < 0.05; Fig. 5B). Thereafter, *dax1* expression declined dramatically as the oocytes degenerate, paralleling with the initiation of spermatogonial proliferation (♀/♂I, ovotestis in I stage) (Friedman ANOVA, *P* < 0.05; Fig. 5B). The *dax1* expression was increased again (♀/♂III), and then decreased to a low level in testis (♂) (Friedman ANOVA, *P* < 0.05; Fig. 5B).

## Discussion

In this study, we isolated the full-length cDNAs of *amh* and *dax1* from *M. albus* and then analyzed and characterized their deduced amino acid sequences. Multiple sequence alignment of Amh showed the C-terminal region shared a high degree of homology among different species owing to the conservatism of the TGF-β family. In most members of this family, the N-terminal region does not have its own biological activity but it is critical to enhance the biological function of the C-terminal region^32^. Sequence analysis of the region between TGF-β and the AMH-N domain suggest that there are two putative cleavage sites (R/K-XX-R/K) in most fish species, including *M. albus*. However, only one cleavage site form in humans and cattle^3^. Phylogenetic analysis of *amh* showed a significant difference between fish and mammal, suggesting a high rate of diversification during the evolution of *amh*^16^. Multiple sequence alignment of Dax1 showed the protein shared a high level of similarity in fish. Four LXXLL-like motifs were found in mammalian DAX1 but only two such motifs have been found in the *M. albus* Dax1 protein, which is highly conservative among teleosts^19^,^30^. The second and fourth LXXLL-like motifs were located in the DNA/RNA/nuclear receptor-binding domain and the ligand-binding domain, respectively. It is documented these LXXLL-like motifs were capable of interacting with *SF-1*^33^,^34^ and other nuclear receptors, including estrogen receptor^22^, androgen receptor^23^ and progestin receptor^35^. Mutation or deletion of these motifs impaired DAX1 repressed activity against *SF1*^36^. In *M. albus*, *D. labrax* and the *Takifugu rubripes* (Pufferfish), the first LXXLL-like motif in the Dax1 protein was followed by a polyglutamine (poly(Q)) stretch. There were seventeen glutamines in *T. rubripes*, thirteen in *D. labrax* and only six in *M. albus*. Glutamine was the most commonly repeated amino acid in eukaryotic proteins. It has been reported that such repeats are part of a general evolutionary mechanism for adding new amino acid sequence^37^. As in mammals, fish have the AF-2 core in the ligand-binding domain and mutation of this residue lowered the frequency of nuclear localization of *sf1*, suggesting an important role of the AF-2 core in nuclear localization processes^21^.

The expression of *M. albus amh* and *dax1* mRNAs was examined in various tissues by RT-PCR. *amh* expression was detected in the gonads (both sexes) but no obvious signal was obtained in other tissues, suggesting *amh* is a related gene in gonadal development in *M. albus*. The similar pattern of expression was found in *O. latipes*^15^ and *P. olivaceus*^13^. It has been well described in mammals that the expression of *Amh* occurs in the Sertoli cells of the testis and the granulosa cells of the ovary^38^,^39^. The high level of *amh* expression was observed in the ovotestis and testis in *M. albus*, and it was also detected in ovary but at a low level. At the same time, in other teleosts, *amh* was expressed in the gonads of one or both sexes, with the highest level in the testis^13^,^14^,^16^,^40^. These findings suggested that regulation of spermatogonial proliferation and differentiation might be one of the common functions of *amh* among vertebrates. In mammals, higher levels of *Dax1* were apparent in different tissues encompassing the gonads but not in the liver or intestine^41^,^42^. Nevertheless, *dax1* mRNA was expressed abundantly in the liver in non-mammalian vertebrates^43^,^44^. The expression of *dax1* has been found in liver and it is also expressed in brain, muscle, spleen, heart, blood, eye and gonads in *M. albus*. *dax1* expressed in the liver might be involved in the regulation of vitellogenesis by interacting with nuclear receptors in *C. auratus* var. Pengze^19^. In *D. rerio*, *dax1* morpholino antisense nucleotide injected embryos exhibited abnormal development in the central nervous systems, indicating it was important for brain development^45^. Therefore, the wide and different expression pattern among vertebrates suggested the function of *dax1* was complex and remained unclear^46^. In addition, *Dax1* has been thought to act as an “anti-testis” gene or a “pro-ovarian” gene during gonadal development^27^, and it was involved in the regulation of ovarian steroidogenesis via inhibition of the transcription of *LRH-1* in a granulosa cell line^47^. *Dax1* was also found to be involved in testis cord organization during development^48^. In the *M. albus* gonad, the expression of *dax1* has been detected in male and female, revealing *dax1* was closely related to gonad development including testis and ovaries.

Next, we used absolute quantitative real-time RT-PCR to examine *amh* and *dax1* expression in different development stages of gonads during sex inversion in *M. albus*. The expression of *amh* has been detected in both testis and ovaries, agreeing with the expression pattern of *amh* in *D. rerio*^14^. In *Anguilla japonica* (Japanese eel)^40^ and *P. olivaceus*^13^ testis, *amh* expression has been detected in Sertoli cells surrounding spermatogonia. In the mammalian fetus, AMH is one of the earliest Sertoli cell-specific proteins expressed by the gonad and it was responsible for the initiation of Müllerian duct regression in the male fetus^49^. *amh* in *A. schlegeli* plays important roles in early testicular^18^. In *M. albus*, the expression of *amh* was up-regulated significantly when gonads developed into the ♀/♂I (ovotestis in I stage), implying *amh* expression was triggered in differentiating Sertoli cells and early testicular development. *amh* expression was continuous up-regulated significantly at ♀/♂II (ovotestis in II stage). It was consistent with the maintenance of *amh* secretion at high levels in the testis, indicating the male development and testis maintained in *M. albus* required sustained release of *amh* in testis. In *D. rerio*^14^ and *D. labrax*^16^, *cyp19a* is down-regulated by *amh*. Conversely, AMH is regulated by other transcription factor, such as SF1, SOX9, DAX1 and WT1^24^,^50^,^51^. Our previous studies investigated the relationship between miRNA-430 and *amh*. The parallel expression levels of *amh* and miRNA-430 were detected in *M. albus*, suggesting that *amh* may be the target gene of miRNA-430^52^. The function of *amh* during gonadal determination and differentiation is regulated by microRNAs in *M. albus*^52^. In addition, the expression of *amh* was detected in granulosa cells in adult female *D. rerio* during the stages of oogenesis. It was discovered first in late stage IB, decreased in early stage III and has not been found in the later stages^14^. Our result in ovaries of *M. albus* agreed with the expression of *amh* in *D. rerio*, which was decreased significantly in ♀IV and hardly detected in ♀V. AMH is an important gene for normal folliculogenesis by regulating the number of follicles^53^. Recent study in mouse found that AMH and FOXL2 synergistically interact to reserve ovarian follicles^54^. This finding supported the role of *amh* in early ovary development. It also approved the important roles of *amh* in natural sex change fish *A. schlegeli* in early ovarian development and late ovarian growth (e.g., vitellogenic oocytes)^18^.

*Dax1* was expressed in all regions of the HPG axis during development. In the ovaries, the granulosa and theca cells stained positive for DAX1. Both Sertoli cells and Leydig cells showed expression of DAX1 in the testis^55^. *Dax1*-deleted mice might have caused ovarian developmental impairment because duplicated *Dax1* caused XY sex inversion, indicating *Dax1* might be an ovary-determination gene. Unexpectedly, the *Dax1*-deficient mice had testis dysgenesis and a spermatogenesis defect in males as the result of disorganized testis cord formation; however, ovarian development was normal^48^,^56^. On the other hand, the expression of DAX1 in Sertoli cells and Leydig cells individually was not sufficient to rescue the pathology, suggesting DAX1 function in other somatic cell types was necessary for proper testicular development, as well as in Sertoli and Leydig cell types^28^,^57^. In females, DAX1 has been detected in various ovarian cell types, especially in granulosa cells, suggesting DAX1 likely had a role in regulation of gene expression during the specific stage of follicular development^21^. It has been postulated DAX1 could be involved in the regulation of some key enzymes by inhibiting *SF1*-mediated transcription in steroid biosynthesis during follicular development^58^, whereas *Dax1*-deficient female mice were normal and fertile with a slight ovarian follicular defect^59^. In this study, *M. albus dax1* was detected in the ovaries and in the ovotestis and testis, and it was present in significantly greater amounts in the ♀IV stage compared to other development stages. These results implied the function of *dax1* in ovary development was complex and not fully understood. Moreover, the expression of *dax1* was significantly decreased when the gonad development entered the ♀/♂I stage, indicating down-regulation of *dax1* was likely one of the factors stimulating testis development. Our previous study demonstrated that *foxl2* may regulate the expression of *cyp19a1a* according to the correlation expression pattern between *foxl2* and *cyp19a1a* in ovary and brain^60^. *Foxl2* knocked-out led to the up-regulation of *Dax1*, *Foxl2* was also regarded as the negative regulator of *Sf1*^61^. The direct interaction between DAX1 and SF1 modulates the various target gene expression, such as aromatase^25^ and MIS^62^. Furthermore, DAX1 also acts as a transactivator for aromatase^25^ and regulates the timing of vitellogenic development in protandrous *A. schlegeli*^63^. Therefore, the cooperative or antagonistical regulation relationship between DAX-1, FOXL2 and SF-1 are complicated. All of them indirectly or directly regulate aromatase expression during the gonadal determination and differentiation^64^.

In summary, full length cDNAs of *amh* and *dax1* were isolated and characterized from the hermaphrodite freshwater fish *M. albus*, and the tissue-specific distribution of these two genes was determined. *amh* is a gonad-related gene expressed mainly in gonads and *dax1* is widely distributed among different tissues. The gonad expression patterns of *amh* and *dax1* were investigated during *M. albus* sex inversion. The amount of *amh* is increased significantly at the ovotestis and maintained at a high level at testis, whereas *dax1* is highly expressed in the ♀IV. It is decreased significantly in the ♀/♂I. Thereafter, its expression was increased again in ♀/♂III, and then decreased to a low level in testis. The information provided in this study will broaden our understanding of the molecular mechanisms underlying gonad development in hermaphrodite fish.

## Methods

### Experimental fish

*M. albus* were obtained from Wuhan in China and cultured in an aquarium for 7 days (34 to 54 cm body length; 50 to 120 g body weight). The fish have been anesthetized by MS-222. Then, they were sacrificed by spinal amputation and segments of the gonads were fixed in Bouin-Holland fluid for histological assessment. The sexual stages refer to our earlier study^65^. The different development stages of gonads and tissues (gonad, blood, muscle, skin, liver, eye, spleen, intestine, kidney, heart and brain) of *M. albus* were frozen in liquid nitrogen and stored at −80 °C. For gonadal analysis: two fishes have been chosen in each stage, and each sample was analyzed in triplicate. For tissue distribution analysis: one kind of tissues from random three fishes was pooled as one sample. All experiments were carried out with the approval from the Institutional Animal Care and Use Committee (IACUC) of Huazhong Agricultural University (Wuhan, China), and also performed in accordance with the International Guiding Principles for Biomedical Research Involving Animals as promulgated by the Society for the Study of Reproduction.

### RNA extraction and reverse transcription-PCR (RT-PCR)

Total RNA was extracted using TRIzol^®^ reagent (Takara, Dalian) according to the manufacturer’s instructions. The quality was assessed by agarose-gel electrophoresis on the basis of the integrity of the 18 S and 28 S rRNA bands and NANODROP 2000c (Thermo Scientific, USA) with an *A*_260mm_/*A*_280mm_ ratio from 1.8 to 2.1. A sample of total RNA of 1 μg was used for reverse transcription with a PrimeScript® RT reagent Kit with gDNA Eraser (Perfect Real Time) (Takara) in a final reaction volume of 20 μL.

### Molecular cloning of *amh* and *dax1*

Partial cDNA fragments of target genes were amplified using degenerate primers (Table 1) designed on the basis of the conserved regions of other species using Primer Premier 5.0 software. PCR was performed with 2 μL of cDNA template in a 25 μL reaction volume containing 0.5 μL of 10 mM each primer and 12.5 μL of Premix Taq (Takara). PCR products were cloned into the PMD-19T vector (Takara) and sequenced at the Beijing Genomics Institute. Gene-specific primers (Table 1) were designed to obtain the 5′ and 3′ ends of each cDNA on the basis of the sequences of these partial fragments following the manufacturer’s instructions for the SMART RACE Kit (Clontech, America).

### Sequence analysis and phylogenetic analyses

The amino acid sequences of Amh and Dax1 were deduced by BioEdit software and aligned with other species using ClustalW. Protein percentage sequence identity was calculated by the MegAlign program of DNAStar software. Phylogenetic trees were constructed using the neighbor-joining (NJ) method of MEGA version 5.05 software and the credibility of each branch was obtained by the use of 1000 bootstrap replicates.

### Tissue distribution of *amh* and *dax1* by RT-PCR

The gene-specific primers (Table 1) were designed to analyze the expression patterns of *amh* and *dax1* among different tissues (brain, muscle, intestine, kidney, spleen, heart, liver, skin, blood, eye and gonads). The 25 μL PCR volume contained 2 μL of 10-fold concentrated cDNA template and the protocol was: heat at 94 °C for 3 min followed by 35 cycles of 94 °C for 30 s, 60 °C for 30 s, 72 °C for 30 s, and a final extension step at 72 °C for 10 min. The *ef1*α (KC011266) and *rpl-17* (KC011267) reference genes were treated for 28 and 25 cycles, respectively. The stability of reference genes have been analyzed in our previous study^65^. All PCR products were electrophoresed in 1% (w/v) agarose gel and DNA was stained with GelRed.

### *amh* and *dax1* patterns of expression during sex inversion of gonads

Absolute quantitative real-time RT-PCR was used to detect the expression of genes among different development stages of gonads. The gene-specific primers used in this experiment were the same as those described above and the specificity of each pair of primers was verified via the only peak of the melt curve. The 25 μL reactive system containing 2 μL of cDNA, 0.5 μL of 10 mM each primer and 12.5 μL of SYBR® Premix Ex Taq™ II (Perfect Real Time) (Takara) and the protocol was: heat at 95 °C for 30 s, followed by 30 cycles of 95 °C for 5 s, 55 °C for 60 s for *amh* or 56 °C for 50 s for *dax1*, and a final extension step at 72 °C for 30 s. A negative control was included in each assay without cDNA and the samples were analyzed in triplicate with a Rotor-Gene Q instrument. The expression of each sample was calculated as copies/ml from the standard curve of serial dilutions.

### Statistical analysis

Two biological replicates were used in each gonadal stage, and each sample was measured three times. All experimental data were reported as mean ± standard error and analyzed by the non-parametric Friedman ANOVA. *P* value less than 0.05 was chosen as the significant level.

## Additional Information

**How to cite this article**: Hu, Q. *et al*. Molecular cloning and characterization of *amh* and *dax1* genes and their expression during sex inversion in rice-field eel *Monopterus albus*. *Sci. Rep*. **5**, 16667; doi: 10.1038/srep16667 (2015).

## Figures and Tables

**Table 1:** Primers used for isolation of genes and real time RT-PCR in *M. albus*.

Primers	Sequence
Deg *amh*-F	5′-TGCAGCCWHACACACAGWCKGTDTG-3′
Deg *amh*-R	5′-RTAKKCCACVGGCACRCAGCA-3′
Deg *dax1*-F	5′-BAAGACBCTGCGSTTCGTGA-3′
Deg *dax1*-R	5′-CTCCAKDAGNACHTCCTCBA-3′
RACE -*amh*F1	5′- CTTGTAAAGAGTGCCCTGAGTCCGTCC -3′
RACE -*amh*F2 (nest)	5′- GCTGTCCTGCTCACATCGCACATTG -3′
RACE- *amh*R1	5′- CAGATGGACCAGAGGGGGCTTAGGG -3′
RACE- *amh*R2 (nest)	5′- GCAGGATGTCACCCAGGAACCGC -3′
RACE- *dax1*F1	5′- TCTGAAAGGAGCTGTGCTGTTCAACCC-3′
RACE- *dax1*F2 (nest)	5′- CACTACATCCAGTCGCTGCGTCGG-3′
RACE -*dax1*R1	5′- CATGGACAGGGCTATGAGCAGCTTG-3′
RACE -*dax1*R2 (nest)	5′- GATCAGCCTGACGTGCTCGTTCAGAG-3′
*ef1*αF (real time)	5′- CGCTGCTGTTTCCTTCGTCC-3′
*ef1*αR (real time)	5′-TTGCGTTCAATCTTCCATCCC-3′
*rpl17*F (real time)	5′-GTTGTAGCGACGGAAAGGGAC-3′
*rpl17*R (real time)	5′-GACTAAATCATGCAAGTCGAGGG-3′
*amh*-F (real time)	5′- AAGGAAGGACAGGGTTTG -3′
*amh*-R (real time)	5′- CATCAGAGGGCAACATTTAC -3′
*dax1*-F (real time)	5′- GCCTATCTGAAAGGAGCTGTGC-3′
*dax1*-R (real time)	5′- TGCTGACGGTCCCTATGACG-3′
